# *trans*-Acting Factors and *cis* Elements Involved in the Human Inactive X Chromosome Organization and Compaction

**DOI:** 10.1155/2021/6683460

**Published:** 2021-05-07

**Authors:** Zhuo Sun, Jinbo Fan, Yufeng Zhao

**Affiliations:** ^1^Institute of Basic Medical Sciences, Xi'an Medical University, No. 1 XinWang Rd, Weiyang District, Xi'an 710021, Shaanxi, China; ^2^Xi'an Key Laboratory of Pathogenic Microorganism and Tumor Immunity, Xi'an Medical University, No. 1 XinWang Rd, Weiyang District, Xi'an 710021, Shaanxi, China

## Abstract

During X chromosome inactivation, many chromatin changes occur on the future inactive X chromosome, including acquisition of a variety of repressive covalent histone modifications, heterochromatin protein associations, and DNA methylation of promoters. Here, we summarize *trans*-acting factors and *cis* elements that have been shown to be involved in the human inactive X chromosome organization and compaction.

## 1. Introduction

X chromosome inactivation (XCI) is the form of dosage compensation used by female cells to balance X-linked gene expression levels between the sexes in mammals [1]. As a result, the inactive X (Xi) is compacted, taking on a rounder and slightly tighter configuration compared to the more flat and extended structure of the active X (Xa) [2]. This compacted structure of the Xi is thought to limit the access of transcription machinery. The Xi is a classic example of developmentally induced heterochromatin, or facultative heterochromatin, which can be readily detected by DNA dyes in human cells [3] as a densely stained mass usually found at the periphery of the nucleus known as the Barr body [4]. Heterochromatin is typically considered transcriptionally silent [5], and heterochromatic regions of the genome are thought of as “condensed” and therefore less accessible to transcriptional machinery.

The initiation of XCI is dependent on a region of the X chromosome known as the X inactivation center (XIC). Inside the XIC, the X-inactive specific transcript (*XIST*) gene encodes for the long noncoding RNA *XIST*, which is expressed from the future Xi, coating it *in cis* [6]. Yin Yang 1 (YY1) is an important transcription activator for *XIST* [7], also serving to tether *XIST* RNA to its own locus [8].

*XIST* induces many epigenetic changes on the Xi, including depletion of euchromatic histone modifications such as histone acetylation [9] and histone H3 dimethylation at lysine 4 (H3K4me2) [10]. Other epigenetic changes are gained including the acquisition of the histone variant macroH2A [11], and the deposition of repressive histone modifications including trimethylation of histone H3 at lysine 9 (H3K9me3) [10] and 27 (H3K27me3) [12]. It is known that H3K27me3 at the Xi is mediated via enhancer of zeste 2 (EZH2) [12], a part of the polycomb repressive complex 2 (PRC2) [13], but it is not known which histone lysine methyltransferase (HMTase) is responsible for Xi H3K9me3 marks. Furthermore, there is DNA CpG island methylation [14] and recruitment of heterochromatin proteins such as heterochromatin protein 1(HP1) [15], structural maintenance of chromosomes flexible hinge domain-containing protein 1 (SMCHD1) [16] and ligand-dependent nuclear receptor-interacting factor 1 (LRIF1), also known as HP1-binding protein (HBiX1) [17].

In addition to chromatin changes, there is also a delay of Xi DNA replication during the S-phase, such that it replicates asynchronously relative to the Xa [18], with the DNA underlying the bands of H3K27me3 replicating during the midlate S-phase and the bands of H3K9me3 replicating after H3K27me3 replication is complete [19]. Collectively, these changes are likely responsible for shutting down most gene expressions originating from the Xi [20].

After the Xi is established, XCI enters the maintenance stage. It is difficult to reverse the effect of gene repression once this stage has been attained, evidenced by the high degree of effort required to reactivate genes on a large scale in somatic cells [21]. Several mechanisms are in place to work synergistically to ensure the repressive state of the Xi. These include continued *XIST* RNA expression, DNA methylation, histone hypoacetylation [22], and the acquisition of macroH2A [23]. DNA methylation is a robust way to keep genes in a repressive state, and the reactivation of genes has been linked with DNA hypomethylation in promoter regions [24]. Additionally, the attainment of DNA methylation may be linked to histone modifications such as H3K9me3, as suggested by a recent study that elucidates the Mbd1-Atf7ip-Setdb1 pathway in the maintenance of XCI [25]. Atf7ip acts synergistically with methyl-DNA binding protein Mbd1 and H3K9 methyltransferase Setdb1, which links DNA methylation with H3K9me3, and this pathway is essential in maintaining the silent state of Xi in somatic cells.

## 2. Proteins Involved in XCI

### 2.1. MacroH2A

MacroH2A is a variant of H2A that has an extensive C terminal tail that makes up two-thirds of the protein [26]. MacroH2A is enriched on the Xi and by immunofluorescence can be seen as an intensely staining structure, called a macrochromatin body, which also coincides with the Barr body in human cells [11]. On a human retinal pigment epithelial cell (RPE1) metaphase Xi chromosome, macroH2A deposition overlaps with H3K27me3 marks, forming an alternating banding pattern with H3K9me3 territories. The accumulation of macroH2A on the Xi occurs in the late stages of Xi establishment.

### 2.2. PRC1 and PRC2

Polycomb complexes PRC1 and PRC2 carry out histone modifications that are integral to the process of XCI. PRC2 complex catalyzes H3K27me3 [27] and PRC1 complex catalyzes ubiquitination of histone H2A at lysine 119 (H2AK119u1) [13]. PRC2 complex' recruitment to *XIST* RNA on the Xi is mediated through the SWI/SNF family helicase/ATPase alpha-thalassemia/mental retardation X-linked (ATRX) [28]. It has been shown that there are around 150 strong binding sites for PRC2 along the Xi, which are mostly within bivalent domains, serving as seeding sites for propagation of PRC2 binding [29]. Bivalent domains are regions of the chromosome exhibiting both H3K27me3-repressive and H3K4me3-active histone marks and are thought to be the signature of developmentally poised genes. PRC2 recruitment to nearby loci mostly within nonbivalent domains is then observed around these seeding sites, laying down H3K27me3 marks in a concentration gradient [29].

There are two pathways by which the PRC1 complex can be recruited to the Xi, either dependent on PRC2 or independent of the presence of PRC2 [13, 30–32]. Early evidence has shown that the PRC2 complex is directly recruited to *XIST* RNA and lays down H3K27me3 [27], which could then be recognized by PRC1 (referred to as canonical PRC1) to lay down H2AK119u1 [13]. More recently, another class of PRC1 complexes called noncanonical PRC1 has been found, whose recruitment to the Xi is independent of the H3K27me3 mark [30]. Examples of noncanonical PRC1 complexes include RING1-YY1-binding protein (RYBP-PRC1) [31] and polycomb group RING finger 3/5-PRC1 (PCGF3/5-PRC1) which can recruit other noncanonical PRC1 complexes and PRC2 complex to establish H3K27me3 modification chromosome-wide on the Xi [32].

The recruitment of polycomb complexes occurs in the early stages of Xi establishment. Polycomb complexes' enrichment on the Xi is readily detectable when *Xist* is induced and the enrichment is lost when *Xist* expression is inhibited [33, 34].

### 2.3. HP1

HP1 is another protein thought to be important for the establishment and maintenance of the Xi heterochromatin. In humans, there are three isoforms of HP1: HP1-alpha, HP1-beta, and HP1-gamma, all of which contain 3 domains: a chromodomain that can recognize and be recruited to the H3K9me3 on the Xi, a hinge domain, and a chromo shadow domain that is important for dimerization and enrichment of HP1 proteins [35–37]. It has been shown that all three isoforms of HP1 can be detected at the human interphase Xi [15]. HP1 can recognize the H3K9me3 modifications and help to maintain the heterochromatin structure and gene silencing on the human Xi and it is generally considered a maintenance factor for the Xi.

### 2.4. hnRNP U/SAF-A and SHARP/SPEN

Although the exact mechanism of how *Xist* mediates the chromosome-wide inactivation and gene silencing is still largely unknown, several factors have been identified to interact with *Xist* RNA and are essential for *Xist*-mediated gene silencing. These factors include hnRNP U/SAF-A [38], SHARP/SPEN [39–42], and hnRNP K [39].

The nuclear matrix binding protein hnRNP U/SAF-A is essential for the anchoring of *Xist* on the Xi [38]. hnRNP (heterogeneous nuclear ribonucleoproteins) are a family of RNA-binding proteins that have important functions in gene transcription regulation. hnRNP U/SAF-A is widely distributed in the nucleus but is concentrated on the Xi [43, 44]. hnRNP U/SAF-A consists of three domains: the DNA-binding SAF domain, SPRY domain, and RNA-binding RGG domain. The SAF and RGG domains are important for the recruitment and localization of *Xist* [38]. Loss of hnRNP U/SAF-A results in delocalization of *Xist* RNA from the X chromosome, and hnRNP U/SAF-A is essential for establishing the Xi during ES cell differentiation [38]. It is also shown that hnRNP U/SAF-A is required for *Xist*-mediated gene silencing [42].

RNA-binding protein SHARP/SPEN is important for *Xist*-mediated gene silencing [39–42], which has recently been verified in preimplantation embryos [45]. SPEN is recruited to enhancers and promoters of active genes on the X chromosome once *Xist* is upregulated and quickly dissociates once the gene silencing is established [45]. *Xist* recruits HDAC3 through interaction with SHARP/SPEN, and HDAC3 removes acetylation modification from histones [39, 40, 42]. SHARP/SPEN expels Pol II [45, 46] and serves as a bridge between *Xist* and transcription machinery/histone modifiers.

### 2.5. SMCHD1 and LRIF1

SMCHD1 was first described in an N-ethyl-N-nitrosourea (ENU) mutagenesis screen to identify genes involved in epigenetic regulation and gene silencing [47]. SMCHD1 belongs to the structural maintenance of chromosome (SMC) domain family of proteins, which also include cohesin and condensin [48], but is different in that its SMC hinge domain is not in the middle of the protein but at the C-terminus. On the Xi, SMCHD1 has been shown to be important for the maintenance phase of silencing [16], heterochromatin compaction [17], and the methylation of CpG islands [49].

SMCHD1 is crucial for both random XCI in the embryo and imprinted XCI in the placenta [16]. The loss of SMCHD1 does not interfere with the accumulation of *Xist* or H3K27me3 modification on the Xi, which suggests that SMCHD1 is not involved in the initiation phase of XCI, but the maintenance phase. This is also implied by the fact that, in interphase nuclei, SMCHD1 is enriched on the Xi [16]. In the absence of SMCHD1, decompaction of the Xi territory is observed [17]. It is suggested that SMCHD1 is enriched on the H3K27me3 territory, whereas LRIF1/HBiX1 is enriched on the H3K9me3 territory through the interaction with HP1, and the interaction between SMCHD1 and LRIF1/HBiX1 brings together the H3K27me3 territory and H3K9me3 territory to form a compacted structure [17]. Notably, PRC2 and H3K27me3 are dispensable for this compaction process, whereas *XIST* is required for the correct localization of SMCHD1 and LRIF1/HBiX1 to the Xi [49, 50].

HBiX1/LRIF1 was first identified as a novel nuclear matrix transcription repressor known as ligand-dependent nuclear receptor-interacting factor 1(LRIF1, RIF1, or C1orf103) [51]. It is an HP1-interacting protein that is enriched on the Xi in interphase nuclei. It has been shown to be essential for the compaction of the Xi chromatin [17, 52]. HBiX1/LRIF1 interacts with SMCHD1 through its coiled-coil domain with the hinge domain on SMCHD1 [17, 52].

### 2.6. SETDB1

SET domain bifurcated 1 (SETDB1), an H3-K9 histone methyltransferase with the highest activity for lysine 9 trimethylation [53, 54], has been shown to be important for establishing H3K9me3 [55] and maintaining gene silencing on the mouse Xi [25]. Recently, it is shown that loss of SETDB1 does not lead to large-scale H3K9me3 changes on the Xi but results in decompaction of the human Xi territory [56]. Although the pathway of SETDB1 recruitment to KRAB-zinc finger proteins through TRIM28 [57] to silence gene expression is well characterized for some autosomal regions [58], it is not known how SETDB1 is recruited to the Xi.

To summarize the process of XCI in regard to the proteins involved and their order of recruitment, hnRNP U/SAF-A is essential for the localization of *Xist* to the future Xi; *Xist* then spreads and coats the whole chromosome. *Xist* recruits polycomb complexes PRC2 and PRC1 and lays down repressive histone modifications. SHARP/SPEN expels RNA polymerase and inhibits transcription. MacroH2A is then recruited and CpG islands are methylated, and at this point, the establishment stage of XCI is concluded. Heterochromatin proteins such as HP1, SMCHD1, LRIF1, and SETDB1 exert their function in the maintenance stage of XCI and work together to maintain the heterochromatin structure and gene silencing on the Xi.

## 3. lncRNAs Involved in X Chromosome Inactivation

The XIC is the minimal region on the X chromosome that is both necessary and sufficient to initiate XCI [59, 60]. The XIC contains some protein-coding genes and some noncoding genes. Essential to the XIC is the region encoding for the *XIST* lncRNA and *TSIX* lncRNA, which is the antisense transcript of *XIST* that can mediate the repression of *XIST* [61].

*XIST* was first discovered to be important for the XCI in mice and humans in 1991 [62–64]. *XIST* lncRNA is 17 kb in length; within *XIST* are several tandem repeat regions that are conserved. The most highly conserved A repeats were found to be essential for gene silencing during XCI [65]. The upregulation of *XIST* expression is the first step of inactivation along the future Xi. The localization of *XIST* to the future Xi is dependent on the transcription factor YY1 and starts with a nucleation center within exon 1 of the *XIST* locus [8]. It has been shown that the spreading of *XIST* follows a stepwise mechanism. It targets gene-rich regions before spreading to intervening gene-poor regions [66]. *XIST* then recruits polycomb proteins PRC1 and PRC2 to set up repressive histone marks for the maintenance of the inactive chromatin structure [27].

While the expression of *XIST* is unique to the Xi, the lncRNA *TSIX* dictates which X chromosome will be the Xa. *TSIX* is the antisense transcript of *XIST* and has an antagonistic role in *XIST* expression. *TSIX* controls *XIST* expression by modifying the chromatin state and DNA methylation of the *XIST* promoter [67, 68]. Before the choice of which X chromosome is going to be the future Xa or Xi, chromosome pairing between the two X chromosomes takes place. The pairing might be important for the redistribution of transcription activators to only allow the transient expression of *TSIX* on the future Xa [69–72].

A few other lncRNAs have been discovered to be important activators for *XIST* expression: RepA [27], Jpx [73, 74], and Ftx [75].

## 4. *cis* Elements Involved in Xi Organization

At interphase in human RPE1 cells, the Xi forms a bipartite structure, with all H3K9me3 bands aggregating together towards the nuclear periphery and the H3K27me3 bands more towards the interior of the nucleus [19]. How and why the Xi arranges itself into these two compartments is not clear. Possible factors that might contribute to this arrangement include the various chromatin proteins that bind to H3K27me3 and H3K9me3 marks, respectively, and self-aggregate. An alternative or additional factor that may contribute to this arrangement is potential DNA folding elements. Several large tandem repeat DNA sequences (TRs) have been identified that are unique to the X chromosome, including the macrosatellite DXZ4, and the TRs X56 and X130. These TRs adopt a Xi-specific euchromatic configuration that is bound by the epigenetic organizer protein CCCTC-binding factor (CTCF) [76]. DXZ4, X56, and X130 have been shown to interact with each other over millions of bases, exclusively from the Xi alleles, potentially acting as epigenetically regulated DNA folding elements [77, 78] forming massive chromosome loops restricted to the Xi [79]. Each TR is located at the intersection between the H3K9me3 and H3K27me3 bands and could contribute to the compartmentalization of the bipartite structure [19].

## 5. Impact of X Chromosome Inactivation on Disease

With the exception of the pseudoautosomal regions located at the tips of Xp and Xq, most genes on the X chromosome have been lost on the Y chromosome. As such, males are hemizygous for most X-linked genes. As a consequence, inheritance of an X-linked recessive mutant allele in males will act as dominant and disease onset will be unavoidable. Females are afforded some protection from X-linked recessive disorders due to XCI.

Because XCI occurs at a multicell stage, and the choice of which X to silence is random and that decision is made independently in each cell, females are a mosaic, where some cells express the mutant allele, and others express the wild-type allele. If the mutant allele impacts cell growth or survival, these cells will be outgrown giving the appearance of skewing toward the wild-type active X. Skewing of XCI occurs naturally, which can affect disease severity. Fabry disease is one example where there is a mutation in the X-linked lysosomal alpha-galactosidase. The peak of the normal distribution represents equal numbers of cells with either the paternal or maternal X chromosome chosen as the Xi. At either tail end of the normal distribution is the phenomenon called skewed XCI, where at one tail end, it might be asymptomatic, but for the other end of the distribution, there is a severe representation of disease. The direction and degree of skewed XCI influence the phenotype and progression of Fabry disease in female patients [80].

XCI serves as a mechanism for balancing the difference in expression between different sexes in mammalian cells, but there is a certain level of escape from gene repression in both normal and disease cells. A recent systematic survey integrating transcriptomes from 449 individuals across 29 tissues has shown that, besides 683 X-linked genes that are consistently inactivated, there is heterogeneity in expression patterns among different individuals and different tissues [81]. X-linked gene reactivation has also been observed in aged tissue [82–84], autoimmune diseases [85], and cancer [86–88]. There is evidence that, in ovarian cancer cells, there is a unique profile of X-linked gene expression and escape, suggesting that XCI may play a role in the development of ovarian cancer [89].

## 6. Concluding Remarks

X chromosome inactivation is the classic model system to study epigenetic questions. What we learn under the X chromosome inactivation context could also be useful to unravel unsolved problems in autosomes and developmental contexts as well. Research and more mechanistic insight for X chromosome inactivation could assist in developing strategies and therapy for X-linked diseases.
